# Effects of age and feeding protocols on the metabolic and physiological condition of unweaned calves during and after long-distance transport

**DOI:** 10.1186/s13620-026-00340-8

**Published:** 2026-03-27

**Authors:** Susanne Siegmann, Luca L. van Dijk, Niamh L. Field, Gearoid Sayers, Katie Sugrue, Cornelis G. van Reenen, Eddie A. M. Bokkers, Muireann Conneely

**Affiliations:** 1 Teagasc, Animal & Grassland Research and Innovation Centre, Moorepark, Fermoy, P61P302 Ireland; 2 Animal Production Systems Group, Wageningen University & Research, P.O. Box 338, Wageningen, 6700 AH The Netherlands; 3 Department of Biological and Pharmaceutical Sciences, Munster Technological University Kerry, Tralee, V92CX88 Ireland; 4 Wageningen Livestock Research, Wageningen University & Research, P.O. Box 338, Wageningen, 6700 AH The Netherlands

**Keywords:** Transportation, Age, Feed deprivation, Biomarkers, Veal, Animal welfare

## Abstract

**Background:**

Long-distance transport and associated extended fasting periods challenge the physiological state of young calves, leading to energy loss, dehydration, and potentially hunger and exhaustion. Older calves with greater body reserves may better withstand fasting, while providing larger volumes of milk replacer pre-transport could help sustain energy balance and hydration. The aim of this study was to investigate how calf age and pre-transport feeding protocols affect the physiological status of unweaned calves during and after long-distance road and ferry transport.

**Results:**

We followed a commercial shipment of 138 male dairy calves from an assembly centre (AC) in Ireland via road and roll-on-roll-off ferry to a lairage in France and then via road to a veal farm in the Netherlands (total transport duration 51 h). Study design was a 2 × 2 factorial with factors calf age (2–3 or 4–5 weeks) and pre-transport feeding protocol (2–4 L of milk replacer). We collected four blood samples (AC, lairage, arrival, Day 7 post-transport) and analysed them for 15 variables indicating energy balance, hydration status, muscle fatigue, and physiological stress responses. Twenty calves were fitted with continuous glucose monitors (CGMs) to measure interstitial glucose from 13 h before to 90 h after departure. Body weight was recorded at AC, lairage, arrival, Day 7 and 21 post-transport. The effects of age and feeding protocol on physiological responses were assessed using linear mixed models with repeated measures and random effects for calf and farm of origin. Younger calves weighed less than older calves overall (49.8 vs. 53.0 kg, *P* = 0.004) and at all sampling moments; they had higher plasma glucose concentrations than older calves overall (4.61 vs. 4.42 mmol/L, *P* = 0.038) and at all sampling moments except lairage, lower sodium concentrations at arrival (139.9 vs. 140.8 mmol/L, *P* = 0.034) and lower chloride concentrations at AC (97.4 vs. 99.0 mmol/L, *P* < 0.001) and lairage (96.6 vs. 97.5 mmol/L, *P* = 0.009). Calves fed 2 L pre-transport had lower CGM glucose readings for two hours after feeding at AC and higher urea concentrations at lairage (4.01 vs. 3.10 mmol/L, *P* < 0.001) than calves fed 4 L.

**Conclusion:**

Although feeding 4 L compared to 2 L before transport had some positive effects on calf energy balance and hydration, our findings suggest that pre-transport feeding has only limited capacity to alleviate the negative impacts of transport and prolonged fasting on the physiological status of calves, especially during multi-day transport. Effects of age on calf resilience were few and inconclusive, and further research should include a larger age range.

## Background

Current EU legislation allows unweaned calves to be transported for up to 19 h without feed, provided that after 9 h, they are given a rest period of 1 h, during which they may stay on the vehicle but must be offered water. Beyond this, a mandatory 12 h rest period including a feed is required before transport can resume [1]. Ireland, one of the largest traders of non-replacement dairy calves within the EU, annually sends approximately 150,000 calves aged 2–5 weeks to mainland Europe, predominantly to the Netherlands and Spain [2, 3]. Given Ireland’s geographical position, these transports routinely exceed a total duration of 50 h, during which calves are only fed at an assembly centre (AC) in Ireland and a control post (lairage) in France [4].

Prolonged fasting during transport is a recognized welfare risk for young calves [5]. To meet their physiological needs, it is advised that unweaned calves consume at least 6 L of milk (replacer) per day [6] and are not fasted for longer than 12 h [7]. However, adherence to these recommendations is often not possible given current transport practices in the EU and worldwide. Dehydration, weight loss, energy depletion, and exhaustion are commonly reported outcomes of long-distance transport (reviewed by [5, 8]) and are accompanied by negative affective states such as hunger and thirst [9, 10]. Physiologically compromised calves are more vulnerable to infectious diseases, and long transport duration is associated with poor health and performance upon arrival at rearing facilities [8, 11].

Optimizing pre-transport nutrition is one potential strategy to mitigate the adverse effects of extended fasting during transport. Research has shown that calves provided with 1.5 L of milk replacer (MR) instead of electrolytes before a 6 h journey maintained higher plasma glucose concentrations and had lower cortisol concentrations upon arrival, indicating that a more nutritious pre-transport diet can mitigate the negative effects of transport on energy balance and the physiological stress response to some extent [12, 13]. In a previous study [14] we found that feeding calves 3 L of MR twice at an Irish assembly centre prior to international transport (the evening before and on the morning of transport) instead of the standard 2 L the morning of transport improved their physiological state during the ferry journey. Calves fed more maintained a better energy balance and showed fewer signs of dehydration during the first section of transport, the ferry journey, but these benefits diminished by the time calves reached their destination in the Netherlands. It is possible that an even larger milk meal before transport could further enhance calves’ ability to cope with prolonged fasting and reduce transport-related physiological changes during and after multi-day transport.

Recently, the European Commission [15] has proposed revisions to legislation governing the transport of unweaned calves, including raising the minimum transport age from 14 to 35 days and introducing a minimum weight requirement of 50 kg. These measures reflect the heightened vulnerability of younger calves to transport-related challenges. Young calves possess limited body fat reserves to cope with energy depletion through fasting [16, 17] and to regulate their body temperature in cold transport conditions [18, 19]. At approximately 2–3 weeks of age calves experience an “immunity gap”, during which maternal antibodies acquired by transfer of passive immunity via colostrum decline before the calf’s active immune system is fully functional, leaving them particularly susceptible disease [20, 21]. Moreover, failure of passive transfer of immunity has been reported to occur disproportionately often in male and non-replacement dairy calves, mostly due to inadequate colostrum management on the farm of birth [22–24]. This increased vulnerability is reflected in the high rates of morbidity and mortality reported in young calves following transport, during and after which calves from multiple source farms are mixed and exposed to novel pathogens [22, 25, 26]. Despite these concerns, only few studies have examined how the age of calves may affect their physiological response to transport [27]. Most of the available research on the impact of transport on calves of different ages is based on experimental or observational data and focuses on young calves aged less than 28 days [28–31]. The paucity of data on older unweaned calves and the scarcity of studies conducted under commercial transport conditions highlight significant knowledge gaps in this area. Moreover, there is a lack of research on the impacts of multi-day transport events involving ferry and road journeys, which are currently common in Irish calf export. Hence, the objective of this study is to investigate the effects of pre-transport feeding protocols and calf age on physiological and metabolic parameters during and following commercial long-distance transportation. We hypothesized that older calves (4–5 weeks) and those receiving a larger milk meal (4 L) prior to transport would demonstrate superior ability to cope with transport, maintaining better physiological homeostasis than younger calves (2–3 weeks) and those fed less MR (2 L). We expected older calves fed more to sustain a positive energy balance for longer, show fewer signs of dehydration, fatigue and physiological stress responses, and lose less body weight (BW) over the course of transport.

## Methods

### Study design and animals

In this study, a commercial shipment of calves was followed from an AC in Wicklow, Ireland, via ferry and road to a veal farm in the Netherlands and for three weeks post transport in March 2023. The experiment was designed as a 2 × 2 factorial with two factors: (1) calf age: 2–3 weeks (“young”: Y) or 4–5 weeks (“older”: O), and (2) pre-transport feeding protocols: calves were fed 2–4 L of MR the morning of transport, resulting in four experimental treatments: Y2L, O2L, Y4L, O4L.

Sample size was calculated using the program G*Power [32], with a focus on one of the main treatment factors, calf age, and one of the main outcomes of interest, plasma glucose concentrations. The expected effect size was calculated from mean plasma glucose concentrations of two groups of calves aged 2–3 (3.6 mmol/L) and 5–6 (3.1 mmol/L) weeks, measured after long-distance transport in a similar previous study [14]. To obtain a power of 0.80 with the significance criterion set at α = 0.05, the minimum sample size needed was 31 calves per treatment. We included 35 calves per treatment (140 in total) to allow for dropouts.

Study animals were selected from calves scheduled for international shipment on the study date. On the evening prior to transport, the exporter provided a list of all calves that were due to depart the following day and that met our predefined inclusion criteria (fit for transport, i.e. alert and responsive, mobile, dry navel, no injuries/wounds, no overt signs of illness such as diarrhoea, bloat, or laboured breathing, assessed by AC staff during intake procedures). From this list of eligible calves, individuals were randomly selected using a random number generator in Microsoft Excel (Microsoft Corp., Redmond, USA). Specifically, each calf was assigned a random number, and calves were selected in ascending order of random number until the required sample size was reached. We had no influence on the exporter’s prior decision regarding which calves were scheduled for shipment. Enrolled calves were then divided evenly into two groups by age, approximately 2–3 weeks and 4–5 weeks old. Within these two groups, calves were blocked by age and breed and randomly assigned to one of two pre-transport feeding treatments (2–4 L of MR) again using a random number generator. Researchers that performed weighing and blood sampling were blinded to treatment allocation. The researcher responsible for assigning treatments handled the animals but was not actively involved in data collection. Staff at the AC, lairage, and veal farm were also blinded to the treatments. After treatment allocation, we attached accelerometers (IceTag, IceRobotics) to the left front legs of 60 calves (15 per treatment) and continuous glucose monitors (CGMs, see section “Measurements”) to the left flanks of 20 calves (5 per treatment). We obtained the subset of 20 CGM calves by again assigning calves to random numbers generated in Excel and selecting the five calves with the lowest number per treatment. We repeated the randomisation process once to ensure the subset was reasonably representative of the larger sample in terms of breed and age (within ± 2 d of the mean age for the whole sample and per treatment; calf BW was not available at this time). Data from the accelerometers is not reported in this article, but their application was considered in the statistical analysis to account for potential confounding effects (see section “Statistical analyses”).

### Transport and housing

#### Pre-transport at AC

**Fig. 1:**
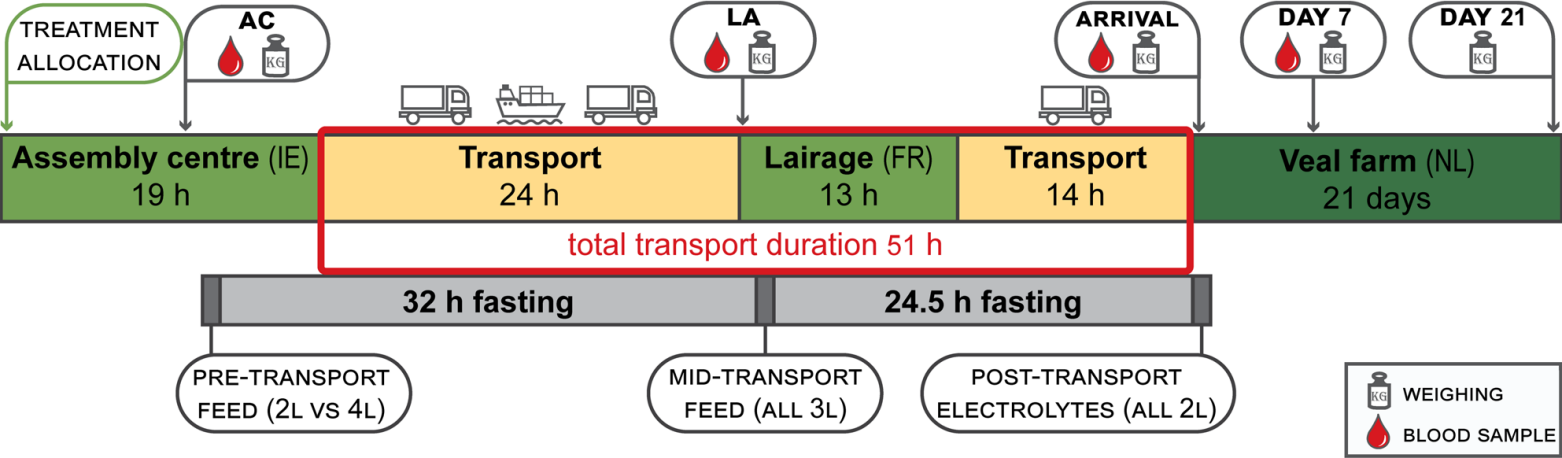
Overview of study and transport schedule including sampling time points (blood sampling and weighing of calves, n = 138), and transport and fasting durations. ‘Feed’ refers to quantities of milk replacer; ‘electrolytes’ refer to an electrolyte mix dissolved in water

An overview of the transport and sampling schedule is presented in (Fig. 1). Calves arrived at the AC from their farms of origin the evening prior to transport (18:00–22:00, all times in UTC/GMT), 20–24 h before departure. Study calves were then selected, randomised, and fitted with CGMs and IceTags (approx. 22:00–01:00). They were housed in eight straw-bedded pens overnight (15–20 calves/pen, space allowance 1.3–1.7 m^2^/calf) with access to water. On the morning of transport (approx. 08:00–11:00), they were weighed and blood sampled (see section “Measurements”) and afterwards fed 2–4 L of MR at 40° C depending on treatment group (MR-AC; 125 g/L) from 10-teat buckets with individual compartments. Details about feeding protocols and feed composition can be found in Table 1. Deviations from the expected drinking volume were recorded. Between feeding and loading them onto a livestock truck, all calves were left to rest for 4.5 h, during which a routine veterinary inspection was conducted by an official veterinarian from the Irish Department of Agriculture, Food, and the Marine to assess their fitness for transport (see fitness criteria detailed above). 

**Table 1:** Feeding protocols and composition of feed types used during and after transport

	Milk replacer			Other	
	MR-AC^a^	MR-L^b^	MR-VF^c^	Electrolyte mix^d^	Muesli^e^
*Feeding protocol*					
Fed on day(s)^g^ (location)	-2 (AC)	-1 (lairage)	1–212 (veal farm)	0 (= arrival) (veal farm)	7–21 (veal farm)
Amount fed per day	2–4 L (treatment-dependent)	3 L	Day 1–6: 3.2 L Day 7–9: 3.4 L Day 10–13: 4.2 L Day 14–16: 5.0 L Day 17–21: 5.4 L	2 L	200 g
Dosage (g/L)	125	90	130	25	-
ME (Mcal/kg)	4.82	4.94	4.91	2.89	3.28
*Composition*					
Crude protein (%)	21	22	21	4.6	13.0
Crude fat (%)	17	19	18.5	0.2	4.0
Crude ash (%)	8.4	8.7	8.2	13.3	4.7
Crude fibre (%)	0.20	0.10	0.05	0.10	8.00
Calcium (%)	0.68	0.78	0.85	0.08	0.68
Phosphorus (%)	0.68	0.65	0.63	0.12	0.40
Sodium (%)	0.75	0.74	0.75	4.09	0.14
Magnesium (%)	-	0.15	-	-	-
*Vitamins (per kg)*					
A 3a672a (IU)	24,990	24,995	25,000	40,000	5500
D3 3a671 (IU)	4005	4005	4000	2540	900
E 3a700 (mg)	150	150	120	-	-
C 3a300 (mg)	-	160	-	-	-
*Trace elements* *(per kg)*					
Fe(Iron(II) Sulphate Monohydrate 3b103 (mg)	78	84	15	-	-
Potassium iodate 3b201 (mg)	-	-	3	-	-
I(Calcium iodate, anhydrous) 3b202 (mg)	0.50	0.50	-	-	0.34
Cu(Copper(II) Sulphate pentahydrate) 3b405 (mg)	9	9	5	8	3
Mn(Manganous Sulphate monohydrate) 3b503 (mg)	29	29	40	138	7
Zn(Zinc oxide) 3b603 (mg)	100	100	-	-	-
Zn(Zinc sulphate monohydrate) 3b605 (mg)	-	-	103	165	17
Se(Sodium selenite) 3b801 (mg)	0.20	0.20	0.25	-	0.10

*Abbreviations:* *MR * milk replacer, *AC *assembly centre, *L *lairage, *VF *veal farm, *ME *metabolisable energy

^a^ Ingredients MR-AC: Whey powder, refined vegetable oils (palm, coconut, rape, soy), wheat gluten, soy protein concentrate, lactose, maltodextrin, caseinate, wheat flour, calcium carbonate, wheat starch, sugar, dextrose, magnesium oxide, propane-1,2-diol

^b^ Ingredients MR-L: Whey powder, refined vegetable oils (palm, coconut, rapeseed), hydrolysed wheat gluten, lactose, wheat starch, dextrose, maltodextrin, caseinate, wheat flour, calcium carbonate, yeast extract, sugar, tallol fatty acids, magnesium oxide, monopotassium phosphate

^c^ Ingredients MR-VF: Whey products, animal fat, skimmed milk powder, coconut oil, wheat protein, wheat starch, animal protein hydrolysate, linseed oil, soy protein concentrate, calcium butyrate

^e^ Ingredients electrolyte mix: Dextrose, plantago powder, wheat flour, whey powder, sodium chloride, sodium bicarbonate, potassium chloride, propane-1,2-diol

^f^ Mixed with 20% chopped wheat straw; ingredients muesli: Maize flakes, barley, maize gluten feed, carob, rapeseed solvent extract, soybean hulls, wheat flakes, palm kernel meal, molasses, dehydrated sugar beet pulp, sweet lupines, alfalfa, maize, wheat middlings, flax hulls, sunflower extraction meal, calcium carbonate, palm oil, straw, monocalcium phosphate

^g^ Days relative to arrival (Day 0)

#### During transport

All study calves and an additional 13 non-study calves were loaded onto a truck via a ramp at an angle of < 20° (the same procedure was followed for all subsequent loading/unloading) and allocated to seven straw-bedded pens. These comprised two 8.25 m^2^ pens on the bottom and top decks, each holding 25–26 calves (space allowance 0.32–0.33 m^2^/calf) and three 5.50 m^2^ pens on the middle deck, each holding 17 calves (space allowance 0.32 m^2^/calf) while ensuring that treatment groups and non-study calves were evenly distributed over the three decks of the truck. Calves were then transported via road and a roll-on-roll-off ferry from Rosslare, Ireland, to a lairage/control post near Cherbourg, France (18:00 to 18:00 the following day). Calves had access to water via rubber teats in the truck during the 21-h ferry journey and during roadside rest stops, but water supply was turned off when driving. Immediately after unloading, calves were again weighed and blood sampled. After sampling, local staff then fed them 3 L of MR at 40° C from teat-buckets regardless of treatment group (MR-L; 90 g/L, Table 1), following the usual feeding protocol of the lairage. During this first part of transport, calves spent 24 h on the truck (of which 21 h aboard the ferry) and were fasted for 32 h. After feeding, all calves from the shipment (including non-study calves) were randomly distributed across four straw-bedded pens (*n* = 35–40 calves/pen, 0.8–0.9 m^2^ space allowance per calf) and allowed to rest with access to water troughs. After 13 h at the lairage, calves were re-loaded and while individual calves were placed on different decks than during the first journey, treatment groups were again evenly distributed over the three decks. The truck left the lairage in the early morning (07:00) and delivered the calves to a veal farm in the province of Gelderland in the Netherlands that same evening (approx. 21:00). Study calves were weighed immediately after unloading and then randomly sorted into individual pens by farm staff. After blood sampling, they received an electrolyte mix dissolved in 2 L of water (25 g/L, Table 1) at approx. 40° C in individual buckets. Calves that struggled to drink from a regular bucket were provided with floating rubber teats for all liquid meals on the veal farm. All calves received their first feed of MR on the farm at approx. 04:30 the following morning. During this second part of transport, calves spent 14 h on the truck, including a 1 h rest stop after 5 h of driving during which calves stayed on the truck but had access to water via rubber teats. They were fasted for 24.5 h between the feed of MR at the lairage and electrolytes at arrival on the veal farm, or 30.5 h between feeds of MR at the lairage and the morning after arrival. From loading at the AC to unloading at the destination farm, calves spent a total of 51 h in transit. Three temperature and humidity sensors (TinyTags, Gemini Data Loggers Ltd., UK), attached to vents along one side of the truck facing outwards (the truck’s interior structure prevented inside placement), recorded median (range) outside temperatures of 15.1 °C (9.9–20.4 °C) and 16.4 °C (10.2–22.4 °C) and median relative humidity of 81% (73–97%) and 53% (40–94%) on the journey from AC to lairage and lairage to veal farm, respectively.

#### Post-transport on farm

On the farm, calves were housed in individual, wooden-slatted pens (100 × 180 cm) in accordance with housing regulations for veal calves in the EU [33]. Treatment groups were randomly distributed over seven rows of pens (14–21 calves/row), while the remaining six rows in the barn were filled with non-study animals. Calves were fed MR twice daily at approx. 45° C from individual buckets (MR-V; 130 g/L, Table 1), starting with approx. 1.6 L per feeding and gradually increasing to approx. 2.7 L over three weeks, which corresponds to standard industry practice. Individual calves received different amounts of MR (1–4 L) from the farmer, who estimated and adjusted feed requirements based on approximate BW. From Day 7 post-arrival, calves were offered muesli (200 g/d, Table 1) mixed with 20% chopped wheat straw. Three TinyTag temperature sensors, attached at calf height to pen structures next to three different rows housing study calves, recorded a median (range) ambient temperature of 16.2 °C (12.6–19.9 °C) and a median relative humidity of 77% (50–86%) in the three weeks post-transport. We weighed and blood sampled calves on Day 7 after arrival and weighed them again on Day 21.

### Measurements

#### Body weight

BW was measured with a calibrated electronic scale (EziWeigh 7i, Tru-Test, Datamars, Switzerland) at five time points: AC, lairage, immediately upon arrival, and on Days 7 and 21 after arrival.

#### Blood samples

We collected four vacutainer tubes of blood (6 ml EDTA, 6 ml heparin, 6 ml sodium fluoride/potassium oxalate, 8.5 ml serum-separating tubes) via jugular venepuncture per calf using a 20-gauge, 1-inch (2.54 cm) BD Vacutainer needle (Becton, Dickinson and Co., Plymouth, UK) at four time points: AC, lairage, immediately upon arrival, and on Day 7 after arrival. EDTA tubes were refrigerated and analysed for red blood cell count (RBC), haemoglobin, and haematocrit concentrations within 12 h of collection at Teagasc Grange (Dunsany, Ireland; AC samples) using the automated haematology analyser ADVIA 2120 (Siemens Healthcare Diagnostics, Eschborn, Germany) or by Rimondia (Elspeet, the Netherlands; all other samples) using the XT-1800i (Sysmex Europe, Norderstedt, Germany). Inter-assay coefficients of variation were 1.1% for RBC, 2.2% for haemoglobin and 1.2% for haematocrit. We centrifuged the remaining tubes at 3000 rpm for 10 min (serum-separating) or 15 min (heparin, glycolytic inhibitor) and froze the separated serum and plasma at − 20 °C until analysis at Teagasc Grange. Plasma glucose, non-esterified fatty acids (NEFA), β-hydroxybutyrate (BHB), urea, total protein (TP), albumin, lactate, creatine kinase (CK) and serum sodium, potassium and corrected chloride concentrations were quantified using the AU480 Chemistry Analyzer with ISE (Beckman Coulter, California, USA; maximum inter-assay coefficient of variation: 10%). A tray of serum-separating tubes was spilled at the AC, and we could not determine electrolyte concentrations (sodium, potassium, corrected chloride) for 22 calves (19 Y2L, two Y4L, one O2L) for this time point. A commercially available ELISA kit (ADI-900-071, Enzo Life Sciences, Brussels, Belgium) was used to determine plasma cortisol concentrations.

#### Continuous glucose monitors

Commercially available CGMs (FreeStyle Libre, Abbot Laboratories, Chicago, USA), which have recently been validated for clinical use in calves [34], were attached to 20 calves at the AC according to the manufacturer’s recommendations. We placed the device on the left flank approx. 10 cm below the spine after clipping the area and wiping it with methylated spirits and secured it with skin-safe superglue. They were left uncovered to allow for easy access and remained on the calves until they detached by themselves or until the end of the study. Subcutaneous interstitial glucose concentrations were measured every 15 min and stored on the CGM for 8 h. To retrieve the data, a paired device (CGM reader or smartphone) was held near the CGM. Due to logistic constraints, i.e. inaccessibility during the ferry journey or during night time on the farm, we were unable to download data every 8 h, resulting in six periods of continuous readings: 13 –2 h before departure from the AC, and 17–25 h (part of the ferry journey), 28–35 h (lairage), 44–54 h (road journey and arrival on veal farm), 58–68 h (day 1 on veal farm), and 80–90 h (day 2 on veal farm) after departure. One CGM reader was accidentally damaged by study personnel and all data for this O2L calf was lost. We also removed all CGM data for one Y4L and one O4L calf from the analysis. Their CGM measurements were continuously low (< 3 mmol/L) after the first few hours of recording and also substantially lower than the glucose concentrations measured in blood samples taken at the same time, which might have been due to defective CGMs or readers, incorrect application of the devices, or the devices coming loose. The final CGM sub-sample included 17 calves (5 x Y2L, 4 x O2L, 4 x Y4L, 4 x O4L).

### Statistical analyses

Statistical analyses were performed using R version 4.4.3 [35]. The study involved a single transport journey, making it the experimental unit. However, for the purpose of statistical analysis, individual calves were treated as independent observational units, acknowledging that true replication at the journey level was not possible. We fitted linear mixed models using the R package ‘lme4’ version 1.1–36 [36] to analyse each variable. The model used to analyse blood variables included calf age (Young = 2–3 weeks, Old = 4–5 weeks), pre-transport feeding protocol (2 L, 4 L), time point (AC, lairage, arrival, Day 7), and all corresponding two- and three-way interactions as fixed effects, and BW before transport as a continuous covariate. Calf was introduced as a random effect to account for repeated measurements, and farm of origin was included as a random effect to account for potential clustering (e.g. similarities in management for calves from the same farm). We initially fitted a model with the additional fixed effects breed (Holstein-Friesian, Friesian crossbreed), the presence of activity sensors and/or glucose monitors to detect potential effects of excess handling or discomfort, and deck of the truck calves were transported on to account for potential environmental differences, but chose to remove these effects for the final model based on model selection using Akaike’s Information Criterion (AIC). For blood variables that showed pre-treatment differences between the feeding groups at AC (sodium, TP, albumin, lactate, CK, cortisol), the corresponding baseline measurement was included as a covariate to account for these initial differences. Baseline values were not used to adjust for initial differences between age groups, as age as a “treatment” is an inherent characteristic that was present before sampling. Age-related differences at baseline are biologically meaningful and of interest as they may influence calves’ ability to cope with transport.

The models used to analyse BW and changes in BW between time points (ΔBW) included an additional fifth time point (Day 21 after arrival) and omitted BW at AC as a covariate to avoid collinearity but were otherwise identical to the one above.

The model for CGM data used the same fixed and random effects structure as described above, but here, ‘time point’ referred to the hours relative to departure from the AC (spanning 13 h before departure to 12 h after arrival at the veal farm, which corresponds to 90 h after departure). CGM glucose concentrations were analysed as hourly means, calculated only for hours in which at least three measurements were available per calf. To assess the representativeness of the CGM subset, we compared baseline characteristics between calves fitted with CGMs and those without using Fisher’s exact test (breed) or t-tests (age, BW, and plasma glucose concentrations at AC).

The residuals of all models were checked for normality, homogeneity of variances, and outliers with the package ‘performance’ version 0.13.0 [37]. The variables BHB, NEFA, urea, CK, and cortisol were log-transformed to meet model assumptions. To assess model fit and the amount of variance explained by the fixed effects, we calculated marginal and conditional R^2^ after Nakagawa and Schielzeth [38] using the package ‘performance’ version 0.13.0 [37]. Fixed effects and interactions were tested for significance (*P* < 0.05) using ANOVA with approximate F-tests from the package ‘car’ version 3.1-3 [39]. For significant effects, we calculated estimated marginal means (EMM) and their pairwise comparisons with the package ‘emmeans’ version 1.11.1 [40]. We additionally performed pairwise comparisons of the four treatment groups at all time points, even when the three-way interaction (age × feeding × time) was not significant. These comparisons were pre-specified based on the study design and biological relevance, allowing us to evaluate how combinations of age and feeding protocol influenced outcomes at key stages of transport. *P*-values for multiple comparisons were corrected with the Tukey adjustment. The mean values and 95% CIs of log-transformed variables were back-transformed and are presented on their original scales.

## Results

### Descriptive statistics

Two O4L calves were excluded from the study after they were declared unfit for transportation during the pre-transport veterinary inspection due to bloating and increased respiratory rate, respectively. Thus, the study included 138 male Holstein-Friesian and Friesian crossbred calves, divided over the four treatments (35 calves each in Y2L, O2L, Y4L, and 33 calves in O4L). Calves originated from 19 different dairy farms, with each farm contributing seven calves on average (= 5% of total n; range 1–14%). Breed composition, mean ages and BW of all study calves, the CGM subset, and the respective treatment groups are listed in Table 2. Calves fitted with CGMs (*n* = 17) did not differ from non-CGM calves (*n* = 121) in breed distribution (0 vs. 17% cross-bred calves, *P* = 0.13), age at AC (26.56 vs. 26.24 d, *P* = 0.82), or BW at AC (48.62 vs. 49.10 kg, *P* = 0.77). Baseline plasma glucose concentrations were numerically higher in CGM calves (4.64 vs. 4.29 mmol/L), but this difference was not statistically significant (*P* = 0.063).

Two calves died after arrival; both were autopsied and found to have died from health complications unrelated to the study (1 x O2L, died on day 12 from MR entering the rumen instead of the abomasum, i.e. ‘rumen drinker’; 1 x Y4L, died on day 18 from mesenteric torsion); for these calves we obtained all blood samples and all but the last BW measurement (Day 21). One calf (O4L) failed to ingest the allotted amount of MR before transport but remained in the study as it consumed 50% more MR than 2 L calves (approx. 3 of 4 L). None of the values from this calf or the two previously mentioned were identified as outliers or influential observations, and all were retained in the dataset.

**Table 2:** Breed, mean ± SD, and range of age and BW at the AC on the day of transport for all calves included in the study, for each treatment (age x feeding protocol), and for subsets equipped with CGMs

Groups	*N*	Breed		Age at AC (d)		BW at AC (kg)	
		FR	FRX	Mean ± SD	Range	Mean ± SD	Range
All calves	138^1^	118	20	27.0 ± 6.0	14–38	49.0 ± 6.0	33.5–69.0
CGM calves	17^2^	17	-	26.2 ± 5.3	14–35	48.6 ± 6.2	33.5–56.0
Treatments							
Y2L	35	31	4	21.7 ± 3.6	14–26	48.4 ± 5.7	33.5–63.5
Y2L CGM	5	5	-	22.0 ± 3.3	17–26	45.0 ± 6.4	33.5–52.5
O2L	35	28	7	31.3 ± 3.5	27–38	50.3 ± 5.7	40.0-61.5
O2L CGM	4^2^	4	-	30.0 ± 1.5	28–32	49.0 ± 6.1	41.0-54.5
Y4L	35	34	1	21.9 ± 3.8	14–26	46.9 ± 5.0	37.5–56.5
Y4L CGM	4^2^	4	-	23.0 ± 5.2	14–26	48.3 ± 2.7	45.5–52.0
O4L	33^1^	25	8	31.5 ± 3.5	27–38	50.6 ± 7.3	38.5–69.0
O4L CGM	4^2^	4	-	31.0 ± 2.6	28–35	54.0 ± 3.7	48.0–56.0

*Abbreviations*: *AC * assembly centre, *FR * Holstein-Friesian, *FRX * Holstein-Friesian crossbreed, *Y *2–3 weeks of age, *O *4–5 weeks of age, *2 L/4L* fed 2–4 L of milk replacer before transport, *CGM * calves equipped with continuous glucose monitors

^1^ Planned sample size was 140 calves (35/treatment); two O4L calves were not fit for transport and removed from the study

^2^ All CGM data from three of initially 20 CGM calves was lost or removed from analysis due to faulty recording

### Model fit and random effects

Conditional R^2^ values for all models showed satisfactory goodness-of-fit, ranging from 31% of variance in the data explained by the model for lactate, to 97% for BW (Table 3). Marginal R^2^ values, which describe the amount of variance explained only by the fixed effects, varied widely between variables. Notably, the models for the haematological variables RBC, haemoglobin, and haematocrit explained > 90% of variability present in the data, but only 10–14% of it was accounted for by the fixed effects, namely the age and feeding treatments and time. Explanatory power of the fixed effects was similarly low for urea and BW. This is reflected in the large variance estimates for the random effects of these variables, indicating considerable variability between calves and farms of origin. Conversely, most of the variability explained by the models for CGM glucose, BHB, NEFA, sodium, potassium, TP, albumin, lactate, and ΔBW can be attributed to the fixed effects, indicating that variability across calves and farms was low. The variability between calves was greater than the variability between their farms of origin for most variables, except for BHB, NEFA, sodium, and ΔBW, where farm was the more prominent source of variability. For lactate, neither calves nor farms showed detectable variability.

**Table 3:** Conditional R^2^ (variance explained by entire model) and marginal R^2^ (variance explained by fixed effects) values from linear mixed models and variance (SD) estimates for the random effects calf (*n* = 138) and farm of origin (*n* = 19) for physiological variables in unweaned calves during transport

Variable	*R* ^2^		Random effects variance	
	conditional	marginal	Calf	Farm of origin
Glucose (plasma)	0.62	0.40	0.11 (0.34)	0.08 (0.28)
Glucose (CGM)	0.81	0.62	0.02 (0.13)	0.00 (0.03)
BHB	0.62	0.58	1.01 (1.12)	1.05 (1.38)
NEFA	0.78	0.75	1.01 (1.17)	1.03 (1.31)
Urea	0.58	0.18	1.12 (1.65)	1.01 (1.13)
Sodium	0.38	0.33	0.01 (0.12)	0.33 (0.58)
Potassium	0.77	0.72	0.03 (0.18)	0.01 (0.08)
Corr. Chloride	0.58	0.34	1.07 (1.03)	0.33 (0.57)
TP	0.81	0.77	1.57 (1.25)	0.21 (0.46)
Albumin	0.75	0.71	0.36 (0.59)	0.07 (0.26)
RBC	0.91	0.10	1.05 (1.02)	0.30 (0.54)
Haemoglobin	0.95	0.10	0.80 (0.90)	0.35 (0.59)
Haematocrit	0.93	0.14	16.5 (4.06)	7.19 (2.68)
Lactate	0.31	0.28	0.00 (0.06)	0.00 (0.05)
CK	0.54	0.34	0.04 (0.20)	0.01 (0.10)
Cortisol	0.66	0.47	0.08 (0.28)	0.01 (0.12)
BW	0.97	0.47	28.99 (5.38)	5.89 (2.43)
ΔBW	0.88	0.88	0.00 (0.01)	0.01 (0.08)

*Abbreviations: CGM * continuous glucose monitoring, *BHB *β-hydroxybutyrate, *NEFA * non-esterified fatty acids, *Corr. Chloride* corrected chloride, *TP * total protein, *RBC *red blood cells, *CK *creatine kinase, *BW* body weight, *ΔBW* differences in body weight over time

### Effects of age, feeding protocol, and time

**Fig. 2:**
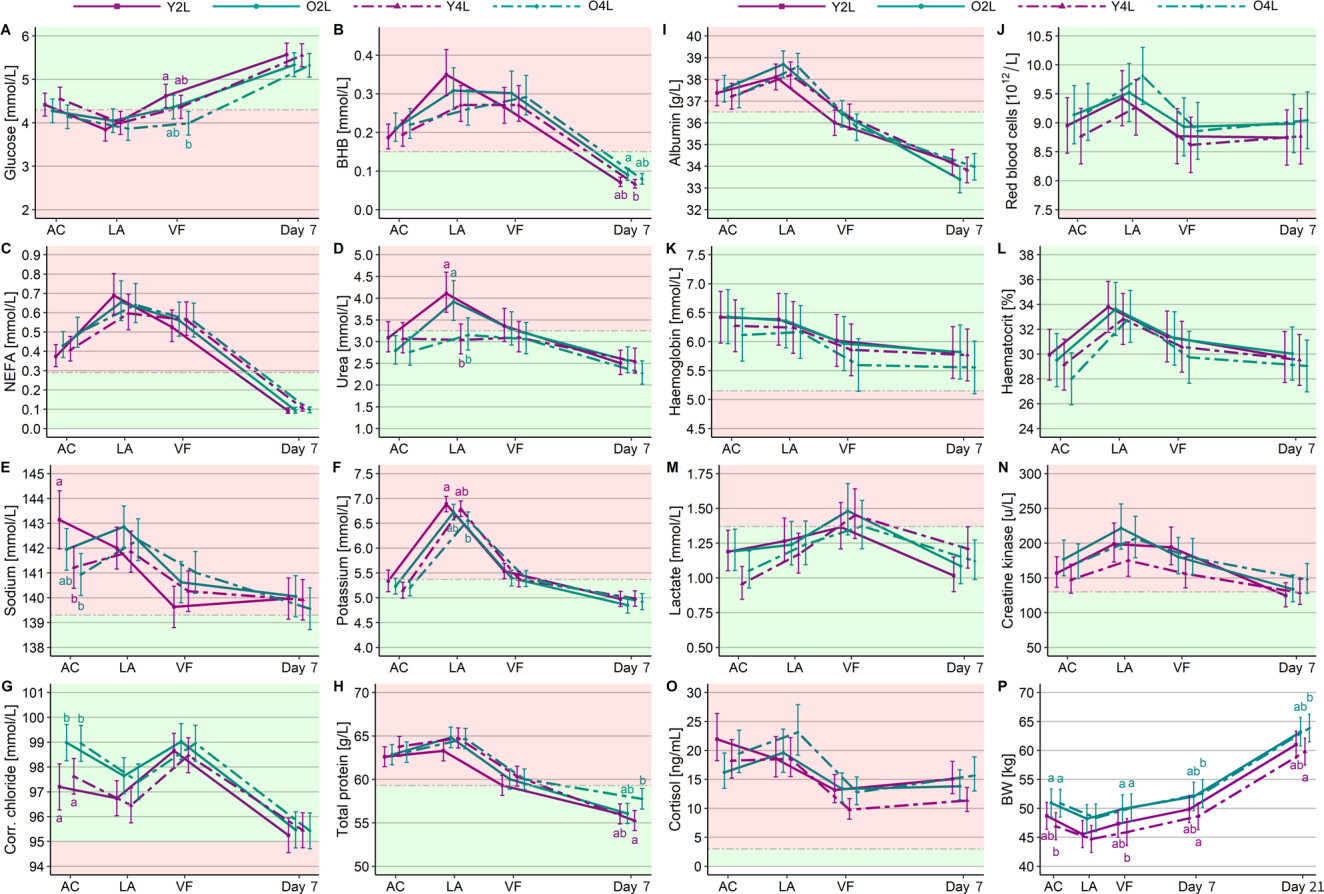
Interactions between treatment (age x pre-transport feeding protocol; Y = 2-3 weeks, O = 4-5 weeks, 2/4 = 2 or 4 L of milk replacer) and time point (AC = assembly centre in Ireland, LA = lairage in France, VF = arrival at veal farm in the Netherlands, Day 7/21 = days after arrival at the veal farm) for glucose (**A**), BHB (**B**), NEFA (**C**), urea (**D**), sodium (**E**), potassium (**F**), corrected chloride (**G**), total protein (**H**), albumin (**I**), red blood cells (**J**), haemoglobin (**K**), haematocrit (**L**), lactate (**M**), creatine kinase (**N**), cortisol (**O**), and body weight (**P**) in unweaned calves (n = 138). Depicted are estimated marginal means ± 95% confidence intervals. Letters indicate significant differences (P < 0.05) between treatments within a sampling time point. Shaded areas represent values within (green) or outside (red) of reference limits for healthy young calves (A, E, F, G, K, L: [41], B, H: [42], C, D, J, N, O: [43], I: [44], M: [45]). Time spans between sampling time points were 31.5 h AC to LA, 25.5 h LA to VF, 8 d VF to Day 7, 14 d Day 7 to Day 21

For each variable, we first describe interactions of age and feeding protocol with time point and significant main effects only when no interaction with time was detected, followed by treatment group effects (age x feeding protocol) over time, depicted in Fig. 2. Reference ranges for each variable, generated from healthy, unfasted calves similar in age and breed to our study calves, are also shown in Fig. 2.

#### Energy balance

There was an interaction between age and time for plasma glucose (*P* = 0.043): younger calves had higher plasma glucose concentrations than older calves at the AC (estimated marginal mean [95% CIs]: 4.51 [4.34, 4.68] vs. 4.16 [3.99. 4.33] mmol/L, *P* = 0.004), arrival on the farm (4.52 [4.35, 4.69] vs. 4.13 [3.96, 4.29] mmol/L, *P* = 0.001), and Day 7 (5.59 [5.42, 5.75] vs. 5.28 [5.11, 5.45] mmol/L, *P* = 0.012), but not at the lairage (3.95 [3.78, 4.11] vs. 3.91 [3.74, 4.08] mmol/L, *P* = 0.765). There were no significant interactions between age and time for BHB (*P* = 0.064) and NEFA (*P* = 0.206) or for feeding protocol and time for plasma glucose (*P* = 0.249), BHB (*P* = 0.099) and NEFA (*P* = 0.232). Differences between the four treatment groups were found for plasma glucose and BHB, but not NEFA (Fig. 2A-C). At the AC, Y4L calves tended to have higher plasma glucose concentrations than O4L calves (*P* = 0.080) and at arrival, Y2L calves had higher plasma glucose concentrations than O4L calves (*P* = 0.001). For BHB, there was a tendency (*P* = 0.052) for Y2L calves to have higher BHB concentrations than O4L calves at the lairage, and on Day 7 after transport, O2L calves had higher BHB concentrations than Y4L calves (*P* = 0.040).

#### CGM glucose

**Fig. 3:**
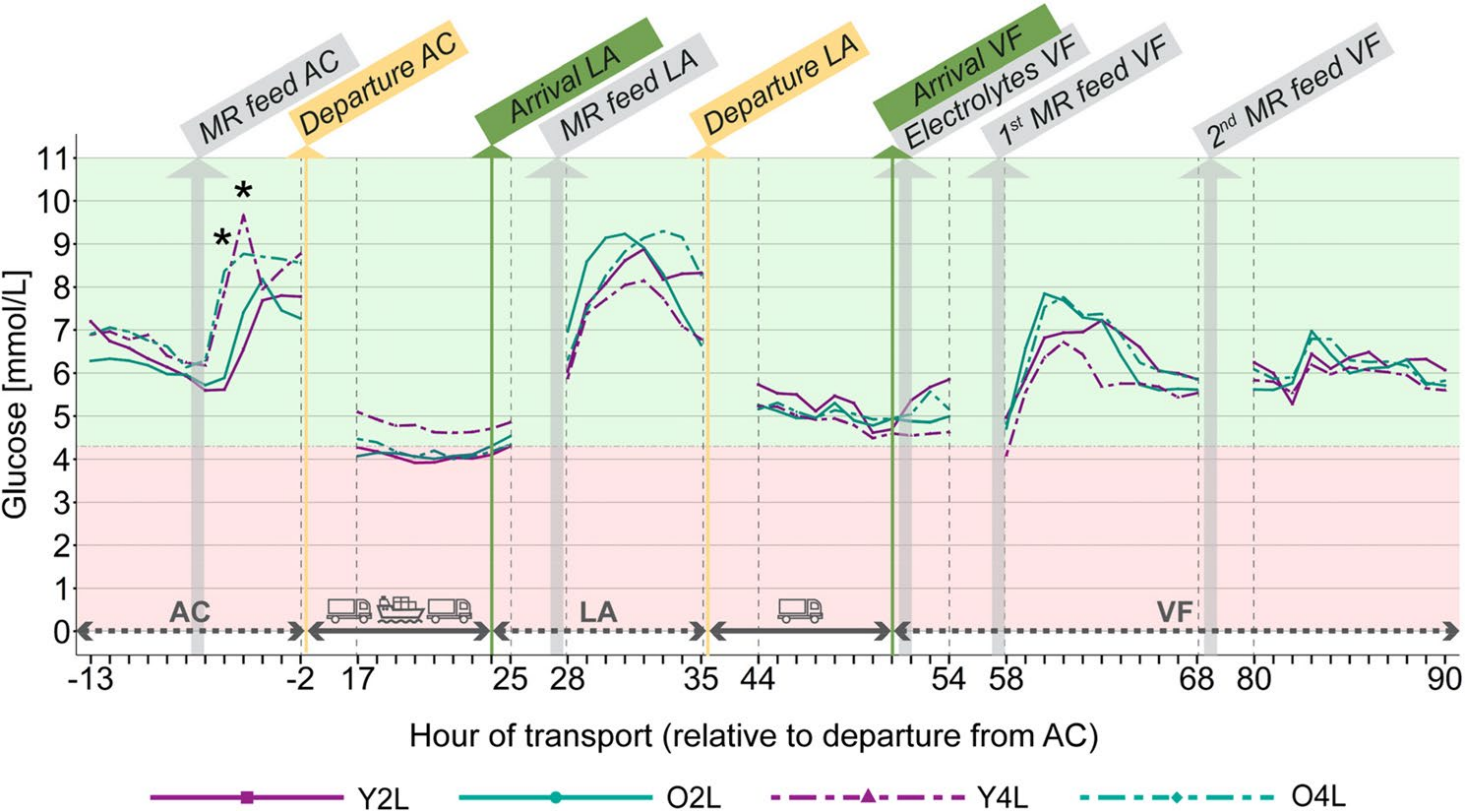
Estimated marginal means of the interactions between calf age (Y = 2–3 weeks, O = 4–5 weeks) and pre-transport feeding protocol (2 L, 4 L of milk replacer) within each hour from 13 h before departure to 90 h after departure from AC for interstitial glucose recorded by continuous glucose monitors in unweaned calves (*n* = 138). Data is not available for all hours as it was not logistically possible to scan monitors every 8 h. AC = assembly centre in Ireland, LA = lairage in France, VF = veal farm in the Netherlands, MR = milk replacer. Asterisks indicate significant differences (*P* < 0.05) between treatments within an hour. Shaded areas represent values within (green) or outside (red) reference limits for plasma glucose in healthy young calves [41]

There was a significant feeding protocol × time interaction for continuous glucose measurements (*P* < 0.001), while the age × time interaction was not significant (*P* = 0.620). Calves fed 4 L had higher CGM glucose concentrations than calves fed 2 L 6 h (8.12 [7.12, 9.27] vs. 5.75 [5.09, 6.49], *P* < 0.001) and 5 h (9.21 [8.07, 10.51] vs. 6.97 [6.17, 7.87], *P* = 0.004) before departure from the AC, or approximately 30–120 min after feeding at the AC. Differences between the treatment groups were found at the same time points (Fig. 3): 6 h before departure, Y4L had higher CGM glucose concentrations than Y2L calves (*P* = 0.041) and O4L had higher CGM glucose concentrations than both O2L (*P* = 0.043) and Y2L calves (*P* = 0.023). The contrast between Y4L and Y2L calves was even more pronounced 5 h before departure (*P* = 0.015).

#### Hydration status

Urea concentrations showed no interaction between age and time (*P* = 0.309). An interaction between feeding protocol and time (*P* < 0.001) showed that urea concentrations were higher for 2 L calves at the lairage (4.01 [3.69, 4.36] vs. 3.10 [2.85, 3.36] 0.13 mmol/L, *P* < 0.001), but not at other time points. Considering treatment groups, 2 L calves had higher urea concentrations at the lairage than 4 L calves regardless of age (Y2L vs. Y4L: *P* < 0.001; Y2L vs. O4L: *P* = 0.006; O2L vs. Y4L: *P* = 0.011; O2L vs. O4L: *P* = 0.028; Fig. 2D).

Age and time interactions were detected for the electrolytes sodium (*P* = 0.021) and corrected chloride (*P* = 0.003) but not for potassium (*P* = 0.399). Younger calves had lower sodium concentrations at arrival (139.9 [139.3, 140.6] vs. 140.8 [140.2, 141.5] mmol/L, *P* = 0.034), and lower corrected chloride concentrations at the AC (97.41 [96.80, 98.03] vs. 99.01 [98.44, 99.51] mmol/L, *P* < 0.001) and lairage (96.60 [96.00, 97.14] vs. 97.50 [97.02, 98.08] mmol/L, *P* = 0.009) than older calves, but no significant differences were found at other time points. We found no interaction between feeding protocol and time for corrected chloride (*P* = 0.660) or potassium (*P* = 0.303). There was an age x time interaction for sodium (*P* = 0.010); however, post hoc the only significant differences between feeding protocols were found at the AC before the feeding treatment was applied. After accounting for this pre-existing variation, the subsequent sampling time points showed no effect of the feeding protocol on sodium. Differences in sodium concentrations between the treatment groups were also only present at the AC (Fig. 2E), when Y2L calves had higher sodium concentrations than Y4L (*P* = 0.035) and O4L calves (*P* = 0.011) and can be largely attributed to pre-treatment differences. Also at the AC, both younger groups had lower corrected chloride concentrations than both older groups (Y2L vs. O2L: *P* = 0.010; Y2L vs. O4L: *P* = 0.011; Y4L vs. O2L: *P* = 0.028; Y4L vs. O4L: *P* = 0.034), and at the lairage, 4YL still tended to have lower corrected chloride concentrations than O2L calves (*P* = 0.067, Fig. 2G). At the lairage, Y2L calves had higher potassium concentrations than O4L calves (*P* = 0.021, Fig. 2F).

Concentrations of TP showed no interactions between age (*P* = 0.199) or feeding protocol and time (*P* = 0.980) but there was a significant three-way interaction between age, feeding protocol and time (*P* = 0.024). The treatment groups differed on Day 7 after transport, when Y4L calves had lower TP concentrations than O4L calves (*P* = 0.014; Fig. 2H). We found no significant interactions between age or feeding protocol and time for albumin (*P* = 0.201, *P* = 0.819) and the haematological variables RBC (*P* = 0.583, *P* = 0.167), haemoglobin (*P* = 0.342, *P* = 0.227) and haematocrit (*P* = 0.070, *P* = 0.276), and no differences were detected between treatment groups at any time point for these parameters (Fig. 2I-L).

#### Muscle fatigue/damage

We found no age x time interaction for lactate (*P* = 0.933) or CK (*P* = 0.488) and no feeding protocol x time interaction for CK (*P* = 0.095). There was an interaction of feeding protocol and time for lactate (*P* = 0.013); however, as for sodium, the only differences between feeding protocols were pre-treatment differences detected at the AC. When comparing the four treatment groups, we detected only tendencies (Fig. 2M-N): Lactate concentrations tended to differ at the AC, with Y4L calves tending to have lower lactate concentrations than both Y2L (*P* = 0.057) and O2L calves (*P* = 0.061). The 4YL calves also tended to have lower CK concentrations than O2L calves at the lairage (*P* = 0.083) and Y2L calves at arrival (*P* = 0.087).

#### Physiological stress (cortisol)

Cortisol showed an interaction of age with time (*P* = 0.024); however, we found no difference between younger and older calves at any of the time points. There was no significant interaction of feeding protocol and time (*P* = 0.073). Possible differences in cortisol concentrations between treatments were detected at the AC, when Y2L calves tended to have higher cortisol concentrations than O2L calves (*P* = 0.086), at arrival, when Y4L calves tended to have lower cortisol concentrations than Y2L (*P* = 0.075) and O2L calves (*P* = 0.067), and 8 days after transport, when Y4L calves tended to have lower cortisol concentrations than O4L calves (*P* = 0.058; Fig. 2O).

#### Body weight

There were no interactions of feeding protocol (*P* = 0.362) or age (*P* = 0.907) and time for BW, but age had an overall effect, with younger animals weighing less than older animals over the entire study period (49.8 [47.9, 51.7] vs. 53.0 [51.0, 55.0] kg, *P* = 0.004). Accordingly, BW differed between treatments at all sampling time points (Fig. 2P), but only Y4L calves had significantly lower BW than O2L calves (AC: *P* = 0.038; LA: *P* = 0.091; VF: *P* = 0.038) and O4L calves (AC: *P* = 0.041; LA: *P* = 0.072; VF: *P* = 0.027) throughout transport. On Days 7 and 21 after transport, Y4L calves continued to be significantly lighter than the O4L calves (Day 7: *P* = 0.044, Day 21: *P* = 0.034).

Table 4 shows ΔBW as well as ADG after transport for the treatment groups. Age and feeding protocol did not influence changes in BW overall, and there were no differences between the treatments. All groups lost BW between AC and lairage and re-gained more than 50% of the loss between lairage and arrival.

**Table 4:** Mean changes in BW (ΔBW) and mean ADG between sampling time points during and until Day 21 after transport in unweaned calves (*n* = 138) per treatment (age: Y = 2–3 weeks, O = 4–5 weeks and pre-transport feeding protocol: 2–4 L of milk replacer)

Study period	Treatment (age x feeding protocol)							
	Y2L		O2L		Y4L		O4L	
	ΔBW (kg)	ADG (kg/d)	ΔBW (kg)	ADG (kg/d)	ΔBW (kg)	ADG (kg/d)	ΔBW (kg)	ADG (kg/d)
AC – LA	-3.1	-	-2.2	-	-2.7	-	-2.5	-
LA – VF	1.8	-	1.2	-	1.7	-	1.7	-
VF – Day 7	2.5	0.36	2.8	0.40	2.2	0.32	2.5	0.35
Day 7–21	11.1	0.79	11.2	0.80	11.2	0.80	11.3	0.81

*Abbreviations*: *AC * assembly centre in Ireland, *LA * lairage in France, *VF *arrival at veal farm in the Netherlands, *Day 7/21* days after arrival at the veal farm

## Discussion

The study investigated the effects of age and pre-transport feeding protocol on the physiological resilience of veal calves subjected to long-distance transport, focusing on markers of energy balance, hydration, physiological stress responses, and muscle fatigue or damage. We proposed that older calves and those provided with a higher volume of MR before transport (4 L) would be able to better withstand transport-related stressors and maintain physiological homeostasis than younger calves or those fed smaller volumes (2 L). Our findings only partially support this hypothesis: There were only few differences between 2 and 3- and 4–5-week-old calves, and while calves fed more before transport showed transient physiological advantages – higher interstitial glucose concentrations before transport and lower urea concentrations at the lairage – these benefits were short-lived and largely overshadowed by the detrimental effects of prolonged fasting and other transport-related challenges on calves’ metabolic status. These results emphasize that long-distance transportation compromises the physiological condition of calves regardless of calf age or pre-transport feeding protocol, highlighting the need for more comprehensive strategies to mitigate its negative impact on calf welfare and subsequent recovery.

### Effects of age

Significant differences in energy balance were observed between younger and older calves. Younger calves exhibited higher plasma glucose concentrations compared to older calves at several time points: at the AC, arrival at the veal farm, and seven days post-arrival. At the AC and arrival, younger calves also maintained plasma glucose concentrations within suggested reference ranges for healthy and unfasted male calves, 11 to 30 d old (4.3–7.4 mmol/L) [41], while older calves fell below it. Seven days post-arrival, both groups were within the reference range. However, during lairage after the ferry journey, no significant age-related differences were noted, and both groups showed plasma glucose concentrations below the reference range. Plasma glucose concentrations in cattle tend to decline with age, so the observed differences may simply be an expression of the aging process and not a physiological response to fasting and transport [42, 43]. This seems particularly plausible since the numerical differences in plasma glucose concentration between the age groups, while statistically significant, seem relatively small, especially when compared to the changes in plasma glucose concentrations between sampling time points.

Contrary to plasma glucose, interstitial glucose measured by CGMs was not affected by calf age, but instead by pre-transport feeding protocol: Calves fed 4 L had higher CGM glucose concentrations than calves fed 2 L in the two hours immediately following feeding at the AC (discussed below). The lack of a feeding protocol effect on plasma glucose concentrations can be explained by the different frequency of measurements: While CGMs measured glucose concentrations every 15 min, blood samples were taken prior to pre-transport feeding, and by the time calves were sampled again at the lairage, the effects of the pre-transport feeding protocol were no longer measurable (by this point, calves had been fasted for 32 h). It is, however, unclear why interstitial glucose concentrations measured by CGMs were not influenced by age, whereas younger calves had higher plasma glucose concentrations throughout the study. One possibility is that age effects were generally less pronounced in the CGM subset. Notably, the overall difference in mean plasma glucose concentrations between young and old calves was smaller in CGM calves (0.05 mmol/L) compared with the full sample (0.19 mmol/L). The origin of this discrepancy remains uncertain, as statistical comparisons confirmed that calves equipped with CGMs did not differ from the rest of the study population in breed distribution (Δ ≈ 17% cross-bred calves), age (Δ < 0.5 d) and BW (Δ < 0.5 kg) before transport, or baseline plasma glucose concentrations (Δ = 0.35 mmol/L). Therefore, it is unlikely that systematic baseline differences between CGM and non-CGM calves explain the reduced age effect observed in the CGM subset. A more plausible explanation is that the smaller CGM subset (*n* = 17) lacked sufficient statistical power to detect subtle age-related differences, as the subset was determined by sensor availability and was not included in the original sample size calculation.

Younger calves also showed fewer signs of dehydration throughout the study period: they had lower corrected chloride concentrations at the AC and lairage, lower sodium concentrations at arrival, and lower TP concentrations on Day 7 after transport. Elevated electrolyte and protein concentrations reflect a reduction in plasma volume and are often reported as the result of long periods without water or food during transport [18, 46, 47]. Despite these differences, both age groups were within normal reference ranges for corrected chloride at the AC and lairage (95–103 mmol/L) [41] and for TP on Day 7 (39.4–59.3 g/L) [42], while sodium concentrations were above the upper reference limit for both age groups the entire study period (> 139.3 mmol/L) [41], suggesting limited biological relevance of the age effect on hydration. Furthermore, as with glucose, it cannot be ruled out that these differences in electrolyte and protein concentrations reflect normal physiological changes of aging [41, 42], however, we would then expect differences for individual variables to appear more consistently.

As expected, 4–5-week-old calves weighed consistently more than 2–3-week-old calves over the whole study period (3 kg on average), but there was no significant difference between age groups in ΔBW during transport and ADG during the first three weeks at the veal farm. Marcato et al. [29] compared the health and welfare of calves aged 14 and 28 days at transport from dairy farms to veal farms in the Netherlands and found that older calves overall showed faster growth, received fewer medical treatments post-transport, and had a lower mortality risk than younger calves. Generally, calf BW at arrival to rearing facilities is known to be one of the most important predictors of future health and performance. Higher BW is associated with decreased mortality [48, 49] and infection risk [25, 50, 51], as well as higher growth rates [52, 53]. Naturally, higher BW correlates with older age, but BW at arrival is also determined by various other factors, including calf breed, health, BW at birth and prior to transport, which is dependent on feeding and management practices on the farm of origin, and the conditions of transport itself such as the season, temperature, duration, feeding protocols and fasting periods [8, 54]. Considering the complex relationships between age and BW, and the abundance of research connecting higher BW to higher resilience to transport-related challenges and health risks, BW may be a more reliable predictor of calves’ ability to cope with transport than age. Nonetheless, older calves are generally less susceptible to infection when mixing with other calves during transport, as they have a more developed active immune system [55].

Overall, age-related differences were limited. Although younger calves had lower BW overall, weight loss during transport and subsequent growth rates did not differ between age groups. Some indicators related to energy balance (plasma, but not interstitial glucose) and hydration status (electrolytes) appeared to be less affected by transport in younger calves; however, these differences were small and unlikely to be biologically meaningful in terms of physiological functioning or welfare. The considerable overall physiological challenge imposed by transport may have overridden some age-related differences. The limited age effects observed may also partly be attributed to the narrow age range of the study sample, with an average difference of only 10 days and no gap between age groups. Future research should explore broader age ranges to better understand the impact of age on transport resilience.

### Effects of feeding protocol

The role of pre-transport nutrition in bolstering calves’ energy reserves and ability to maintain homeostasis during fasting periods is well documented. Several studies have shown that calves fed electrolyte solutions before transport [12, 56, 57] or during rest-stops [58] instead of more nutrient- and energy-rich MR are more likely to rely on metabolising body fat reserves to maintain energy balance and lose more weight during subsequent transport. In the present study, the positive effects of a larger pre-transport milk meal were demonstrated in calves’ energy status immediately following feeding, and urea concentrations at the lairage and upon arrival. Calves fed 4 L of MR before transport had higher interstitial glucose concentrations for 2 h post-feeding, which was to be expected, as plasma glucose in unweaned Holstein calves peaks around 30 min after feeding [59], and a slight time lag between plasma and CGM-recorded interstitial glucose concentrations has been reported in several species [60, 61]. Calves fed more also had significantly lower concentrations of urea at the lairage than calves fed less and remained within normal reference ranges throughout transport (2.5–3.25 mmol/L) [43], while 2 L calves exceeded the upper reference limit for urea at the lairage and at arrival at the veal farm. Elevated urea concentrations are an indicator of dehydration, but also protein catabolism as a response to prolonged fasting [62].

However, most physiological markers were unaffected by pre-transport feeding protocol, underscoring the limited efficacy of a single large milk meal prior to departure. Positive effects on energy balance were absent or short-lived: There were no differences between feeding treatments in BHB or NEFA concentrations at any stage of transport. In fact, BHB (> 0.15 mmol/L) [43] and NEFA concentrations (> 0.29 mmol/L) [42] were elevated above upper reference limits throughout transport in all treatments, indicating the mobilisation of body fat for energy gain in the absence of food [5]. CGM measurements showed that interstitial glucose rapidly declined after the initial post-feeding peak at the AC. By hour 17 of transport, while on the ferry, all treatments except Y4L were hypoglycaemic. Calves may have crossed the lower reference limit for plasma glucose even earlier (< 4.3 mmol/L) [41], but CGM data was not recorded between hours 2–17 of transport. (Although this reference range applies to plasma glucose, Brobst et al. [34] found that CGM-measured interstitial glucose differs from plasma glucose by only 0.1 mmol/L in hypoglycaemic calves.)

In our previous research [14], calves were provided an extra meal of 3 L of MR on the evening before transport in addition to 3 L of MR on the morning of transport, to reduce the fasting period at the AC. In contrast, in the current study we offered calves a single 4 L milk meal on the morning of transport, but the latter approach had fewer positive effects on calf energy balance and hydration status. While calves fed the additional milk meal had higher plasma glucose concentrations and lower NEFA and BHB as well as lower concentrations of electrolytes and urea at the lairage compared to the standard pre-transport feeding protocol (2 L MR the morning of transport), the positive effect of the single 4 L meal in the present study was mainly expressed in lower urea concentrations at the lairage. Even so, it should be noted that the positive effects of both approaches only lasted until arrival at the lairage, and calves in both studies still exceeded normal reference limits for many variables throughout transport. Although a direct comparison of these two pre-transport feeding strategies is not available, findings from the two studies—conducted along the same route with comparable fasting and shipping durations—suggest that feeding an additional meal at the AC may better mitigate the physiological impact of transportation than providing a single, larger milk meal beforehand. However, substantial variability in metabolic outcomes has been observed between shipments of calves transported along the same route under similar conditions [47]. Thus, a direct, controlled comparison of these feeding strategies is needed to confirm this hypothesis and substantiate the observed trends.

The negative welfare impact of the long ferry journeys without opportunities to unload calves for feeding highlights the need, if they continue, to explore alternative methods of transport (e.g., livestock vessels, plane transport) or technical innovations such as delivering warm MR to calves while the remain loaded on trucks. Other practical approaches to shorten long fasting periods include feeding calves closer to departure and providing a second feed during the 13 h rest stop at the lairage. Feeding calves 1–2 h before departure would shorten the second leg of feed deprivation substantially (up to 10–11 h), allowing calves to maintain a better energy balance until they reach their destination farm. Evidence from transport studies [58, 63, 64] shows that feeding young calves within a shorter interval (e.g., within 1 h of loading) is generally well tolerated, although some guidelines recommend feeding calves at least 3 h before departure to allow for digestion and minimize risk of diarrhoea [7, 65].

Moreover, the study’s findings call attention to the limitations of feeding electrolyte solution upon arrival at the destination farm. While electrolytes are commonly administered to alleviate dehydration, CGM data indicated that they provided minimal improvement in energy recovery: At least one treatment group (Y4L) fell below the reference limit for plasma glucose concentrations in the span between arrival and the first milk meal the following morning, despite receiving electrolyte solution (CGM data is missing for most of this time span, but data trends indicate similar trajectories for all groups). Bajus et al. [58] similarly reported suboptimal recovery (more fat mobilisation, worse energy balance) when calves were fed electrolytes compared to MR after transport at a rest stop, and Pempek et al. [66] found that feeding calves electrolyte solution for 0–3 days post-transport (additionally to MR) had very limited positive effects on re-hydration and energy recovery. These findings underscore the need to prioritize feeding MR upon arrival. Overall, this study reaffirms the importance of optimizing not only pre-transport feeding strategies to prepare calves for transportation but also feeding practices during rest stops - particularly when transport durations are long and span several days - and immediately and in the days following transport.

### Comparison with reference ranges

Aside from the variables already described above, most blood variables as well as ΔBW and ADG were unaffected by age or feeding protocol: indicators of energy status and fat mobilisation (BHB, NEFA), muscle fatigue/damage (CK, lactate), physiological stress (cortisol), and many hydration-related variables (potassium, RBC, haemoglobin, haematocrit, albumin) showed no treatment effects, but changed significantly in response to transport. Following the trajectory of variable concentrations across transport, and comparing these values to reference ranges of healthy, unfasted calves similar in age and breed to our study calves (see Fig. 2), we find that calves were already physiologically compromised at the AC, with 7 of 15 blood variables outside the reference ranges for all treatments: Older calves were hypoglycaemic, fat mobilisation was activated (high NEFA and BHB concentrations), and several markers of hydration were elevated. After the ferry journey, their physiological state worsened (9/15 variables outside reference limits), indicating progressive energy depletion and dehydration. At arrival, 8 of 15 variables remained outside reference limits, but had largely returned to pre-transport concentrations, and by Day 7 after arrival, most had normalised. Exceptions to this pattern include corrected chloride and the haematological variables (RBC, haemoglobin, haematocrit), which were inside reference limits at all times, whereas sodium, CK, and cortisol exceeded upper reference limits throughout transport [41, 43]. While transport represents an exceptional and relatively short-term situation in these calves’ lives, and some physiological adaptation is to be expected, our findings show that transport, and specifically the long fasting period, have detrimental effects on calf metabolism regardless of calf age and pre-transport feeding protocol.

### Effects of calf and farm of origin

The contribution of calf- and farm-level effects varied considerably across physiological parameters. Most of the explained variation in haematological variables (RBC, haemoglobin, haematocrit) and BW could be attributed to calf and farm effects rather than to calf age or pre-transport feeding protocol. Farm-to-farm differences were more important than individual calf differences for BHB, NEFA, sodium, and ΔBW, suggesting that management and feeding practices on the farm of origin shape calves’ response to transportation mainly in terms of BW and energy balance (BHB, NEFA). The feeding regimen on the dairy farm, e.g., the frequency, amount, and type of feed (MR or electrolytes), and the time between the last meal and departure, naturally play a large part in the calves’ growth and ability to maintain their energy balance at the AC and during later transport [67]. Even so, the majority of observed physiological markers showed larger differences between individual calves than between farms, and farm of origin had almost no effect on indicators of physiological stress (cortisol) and muscle fatigue/damage (lactate, CK). Farm effects may be more influential when considering health and mortality outcomes. Farm-specific factors that can affect calf health and susceptibility to infection include colostrum management, access to veterinary care and vaccinations, and housing conditions including hygiene practices [57, 68, 69]. While the importance of fixed and random effects is not directly comparable, our results suggest that calf- and farm of origin-specific factors play a substantial role in calves’ ability to cope with transport. Future research should explore conditions or practices on the farms of origin that impact calves’ physiological status and resilience to transport challenges, as well as calf-specific factors (e.g. sex, breed, health status).

### Strengths and limitations

A key limitation of our study is the lack of replication at the journey level. All calves were transported as part of a single shipment, meaning the journey was the true experimental unit. However, due to practical constraints, we treated individual calves as independent observational units in the statistical models. While we accounted for clustering by including farm of origin as a random effect, the lack of independent journey replicates limits the generalisability of our findings and increases the risk of pseudoreplication. It is likely that calf responses were influenced by shared environmental factors, such as truck and driver, temperature, weather, and journey length, which can vary widely between shipments even on similar transport routes. Future studies should aim to replicate treatments across multiple independent journeys to strengthen inference.

The commercial setting of this study enhances the external reliability of our findings and provided valuable insights into the practicality of implementing the feeding treatments and utilizing sensors under real-world conditions. However, the reliance on commercially transported calves introduced several limitations, as detailed information regarding pre-transport management practices at their farms of origin was not available. Baseline physiological parameters measured at the AC could have been influenced by potential confounding factors such as the unknown duration of calves’ transport to the assembly centre and the type, amount and timing of their last feeding at the farm of origin. Physiological parameters during the second leg of transport may also have been affected by feed intake at the lairage, which was not recorded at the individual level. Similarly, the veal farmer feeding calves different amounts of MR based on their estimated BW may have had confounding effects on data recorded on Day 7 after transport. Another consequence of following commercial shipments was that our study was limited to the calves present at the AC the day before transport, which produced a study sample with a relatively narrow age range. This may have minimized detectable differences between age groups. Consequently, we found only few effects of age on physiological status, whereas previous research suggested that older calves are better able to cope with transport challenges [29, 30, 64]. Including older calves at around 6–8 weeks of age that possess a further developed active immune system and larger body reserves [55] would provide a broader perspective on age-related resilience to transport-related challenges.

A strength of the study was the use of continuous glucose monitoring to obtain insights into the physiological responses of calves during transport. The application of CGMs provided a minimally invasive method to track glucose concentrations more closely, thereby reducing reliance on repeated blood sampling via venepuncture, which is a stressful event for calves. The clinical use of CGMs has been previously validated in calves [34], but this study additionally shows that these sensors can be used in a transport setting with minimal risk of injury to the calves or loss or destruction of the devices. Two of 20 CGMs recorded faulty data, which might have been avoided by covering the CGMs with adhesive bandage to prevent them from detaching from the skin [34]. However, the generalizability of findings related to glucose dynamics during transportation from this study are limited because of the small sample size for CGMs (*n* = 17; 4 or 5 calves per treatment), compounded by inaccessibility of calves on the truck and resulting data gaps during key transport stages (i.e., on the ferry, during road transport). We encourage the integration of non-invasive monitoring techniques, such as CGMs or accelerometers, in future studies, but practical obstacles still need to be addressed for optimal use in real-life conditions.

## Conclusions

Positive effects of a 4-L- compared to a 2-L-milk meal before long-distance transport on the metabolic status of unweaned calves were limited to a short-lived increase in interstitial glucose concentrations and lower blood urea concentrations at the mid-journey lairage and arrival at the destination farm. Almost all blood variables indicating energy balance, dehydration, muscle fatigue or damage, and physiological stress responses were outside normal reference ranges for healthy calves at numerous time points during transport, but most notably following ferry transport. Based on these results, we reason that pre-transport feeding protocols have a limited capacity to improve the physiological condition of young calves over long transport periods, and recommend exploring other aspects of diet surrounding transport, such as feeding practices on the farm of origin, on the truck during the ferry crossing, during mid-journey rest stops, and upon arrival at the rearing facility. We found no conclusive evidence of significant differences between 2 and 3- and 4–5-week-old calves in coping with challenges to their metabolic state during transport; however, the overall detrimental impact of transport on the calves’ physiological condition may have masked possible subtle effects of age. Body weight, while associated with age, may be a more impactful parameter for calves’ capacity to cope with transport. However, this study did not cover health outcomes and performance beyond three weeks at the veal farm, which might be more affected by age. There is a need for research involving calves older than 28 days and including health and production parameters over the entire fattening period.

## Data Availability

The datasets used and/or analysed during the current study are available from the corresponding author on reasonable request.
